# Effectiveness of moxibustion therapy in the treatment of urticaria: A protocol for a systematic review and meta-analysis

**DOI:** 10.1097/MD.0000000000023481

**Published:** 2020-12-04

**Authors:** Gen Deng, Wenguo Ye, Qun Wan, Jinlong Wang

**Affiliations:** a College of Acupuncture and Massage, Jiangxi University of Traditional Chinese Medicine; b Affiliated Hospital of Jiangxi University of Traditional Chinese Medicine, China.

**Keywords:** moxibustion, systematic evaluation and meta-analysis, urticaria

## Abstract

**Background:**

Urticaria is a common skin disease in clinic. The main clinical symptoms are sudden attack, various forms, different sizes of wind, and erythema, accompanied by varying degrees of itching. At present, antihistamines, non-specific antiallergic agents, or glucocorticoids are the main treatment, with some side effects and adverse reactions. Moxibustion therapy has shown strong advantages in the treatment of urticaria, and the curative effect is accurate. Therefore, this paper will carry out a systematic evaluation and meta-analysis of the efficacy and safety of moxibustion in the treatment of urticaria.

**Methods:**

Eight electronic databases will be searched, including PubMed, Excerpta Medica Database, Web of Science, Cochrane Library, the China National Knowledge Infrastructure (CNKI), Chinese Science and Technology Periodical Database (VIP), Wanfang Database (WF), and Chinese Biomedical Literature Database (CBM). We will search above electronic databases from the beginning to October 2020, without any language restriction, but involving only the human subjects. Clinical efficacy, including total effective rate or cure rate, and recurrence rate will be accepted as the primary outcomes. The itch level, number of clusters, size of clusters, and laboratory test results will be used as secondary outcomes. The Cochrane Handbook of Systematic Review (5.3.0) RCT risk assessment tool will be used to evaluate the risk of bias by 2 independent researchers.

**Results:**

After the completion of this study, the results will be reported, so it is not possible to give accurate results at present.

**Conclusions:**

The results of this study will provide reliable evidence for the efficacy and safety of moxibustion in the treatment of urticaria.

**INPLASY Registration number:**

INPLASY2020100040.

## 1 Introduction

Urticaria, also known as “measles block”, is a relatively common clinical skin disease, is due to the skin, mucous membrane small blood vessel dilate and permeability increase and a local edema reaction, clinical symptoms are mainly manifested as a sudden attack, form diversity, size of the wind, and erythma, accompanied by different degrees of pruritus.^[1]^ The pathogenesis of urticaria complex, most scholars believe that the disease is associated with allergy, the immune system function in shift, can happen at any age stage, especially the age in 20–40 young women between the age of incidence of a disease is higher, the prevalence of around is also different, may be related to region, the influence of environment and population difference.^[2]^ With the change of peoples lifestyle and living environment, the incidence of disease has been increasing, and it also shows an overall rising trend in the whole world.^[3]^ Urticaria not only affects patients quality of life, but also leads to a higher incidence of anxiety and depression than the normal population.^[4]^ Modern medicine mainly uses antihistamines, non-specific antiallergic preparations, or glucocorticoids in the treatment of urticaria, which is effective in the short-term. However, it is easy to relapse after drug withdrawal, with large adverse reactions and dependence.^[5]^ Besides, the long-term efficacy is not ideal, so it is not suitable for long-term repeated use.^[2]^ Moxibustion therapy is a green, safe, simple, and effective external therapy, which has been proved by a large number of literatures. It is widely used in the treatment of various diseases in clinical practice.^[6,7]^ In recent years, literatures on moxibustion therapy for urticaria have been increasing year by year. However, there is still a lack of systematic evaluation on the efficacy and safety of moxibustion therapy for urticaria in clinical practice. Therefore, the effectiveness and safety of moxibustion in the treatment of urticaria will be systematically evaluated and meta-analyzed in this paper.

## 2 Methods

### 2.1 Inclusion criteria for study selection

#### 2.1.1 Types of studies

Clinical randomized controlled trials (RCTs) containing moxibustion for urticaria were included, with no limitation of language and publication status.

#### 2.1.2 Types of participants

There are clear and recognized diagnostic criteria and efficacy criteria, and all patients are diagnosed as urticaria, regardless of gender, age and origin of the case.

#### 2.1.3 Types of interventions

##### 2.1.3.1 Experimental interventions

Moxibustion therapy will include all therapies using any type of moxibustion, such as indirect moxibustion, direct moxibustion, heat-sensitive moxi-bustion, and so on. Mixed therapies based on moxibustion will also be included.

##### 2.1.3.2 Control interventions

The control group will receive one of the following treatment methods: conventional pharma-cological therapy, no treatment, and placebo. RCTs comparing different types of moxibustion therapy will be excluded.

#### 2.1.4 Types of outcome measures

##### 2.1.4.1 Primary outcome

Clinical efficacy, including total effective rate or cure rate, and recurrence rate will be accepted as the primary outcomes.

##### 2.1.4.2 Secondary outcomes

The itch level, number of clusters, size of clusters, and laboratory test results will be used as secondary outcomes.

### 2.2 Exclusion criteria

Non-randomized controlled trials; no exact diagnostic scale or therapeutic scale; non-moxibustion was the main treatment in the experimental group, and moxibustion therapy was found in the control group. Repeated literature; Theory and review literature; Animal experiments; Nursing research.

### 2.3 The retrieval methods and strategies of this study

#### 2.3.1 Electronic database retrieval

Eight electronic databases will be searched, including PubMed, Excerpta Medica Database (Embase), Web of Science, Cochrane Library, the China National Knowledge Infrastructure (CNKI), Chinese Science and Technology Periodical Database (VIP), Wanfang Database (WF), and Chinese Biomedical Literature Database (CBM). We will search above electronic databases from the beginning to October 2020, without any language restriction, but involving only the human subjects. According to the principle of PICOS, we will search the relevant literature by combining subject words with free words, search terms consist of disease (urticaria or addiction rash or wind mass) and intervention (moxibustion or moxa or moxabustion or cauterize or mugwort) and research types (randomized controlled trial or controlled clinical trial or random trials or RCT). The Cochrane Library search strategy is shown in Table 1.

**Table 1:** Retrieval strategies in Cochrane library.

ID	Search
#1	MeSH descriptor: [Urticaria] explode all trees
#2	(Urticarias): ti, ab, kw OR (Hives): ti, ab, kw
#3	#1 OR #2
#4	MeSH descriptor: [Moxibustion] explode all trees
#5	(Moxabustion): ti, ab, kw OR (moxa): ti, ab, kw OR (Cauterize): ti, ab, kw OR (mugwort): ti, ab, kw
#6	#4 OR #5
#7	#3 AND #6

Ab = abstract, kw = keyword, MeSH = medical subject headings, Ti = title.

#### 2.3.2 Searching other resources

This study will combine manual retrieval of literature resource database to search relevant conference papers that meet the inclusion criteria. In addition, the grey literature, as well as ongoing and recently completed studies, will be searched on Clinicaltrials.gov.

### 2.4 Data extraction and management

#### 2.4.1 Literature inclusion and data extraction

**Figure 1:**
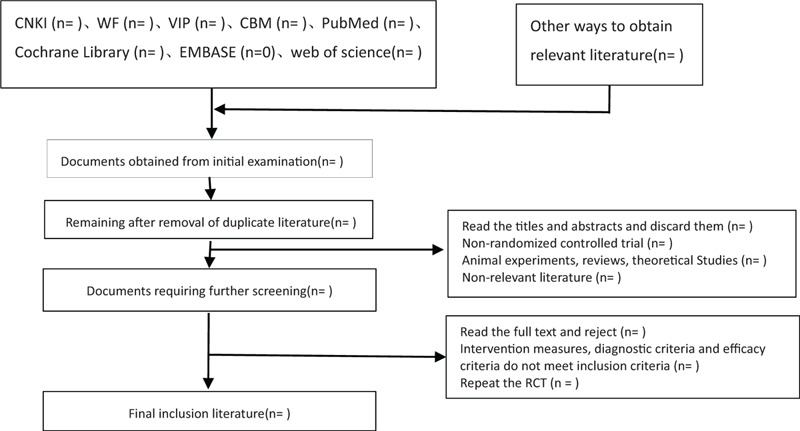
Flow chart of literature incorporation. This flowchart is about the inclusion and exclusion of the literature in order to obtain the final document.

The 2 researchers independently read the title and abstract of the literature we obtained, read the full text of the trials that might meet the inclusion criteria to determine whether the inclusion criteria were truly met, and discussed the conflicting literatures or let the third researcher decide whether to include them. Two researchers independently extracted data from the included studies, including study design, intervention measures, and methods, measurement indicators, results, methodological contents such as hidden grouping and blind method, etc., and a third evaluator checked the consistency of the data. If the required information is incomplete, we will contact the original author for the required data. The inclusion process of this study will be carried out as shown in Figure 1.

#### 2.4.2 Methodological quality evaluation

The Cochrane Handbook of Systematic Review (5.3.0) RCT risk assessment tool will be used to evaluate the risk of bias by 2 independent researchers, including:

1. The method of random sequence generation;
2. Allocation hiding;
3. Whether the subjects and the implementer of the treatment plan should be blinded;
4. Blind method shall be applied to evaluators;
5. Integrity of result data;
6. Selective reporting of results;
7. Other bias.

According to the results of each study that meets the inclusion criteria, according to the above 7 items, objectively judge that each study is high-risk or low-risk or unclear (no relevant information or uncertainty of bias is mentioned in the literature) and explain the reasons. If there are any differences in the above quality evaluation and data extraction process, 2 people shall discuss and resolve the differences or consult the third reviewer to deal with the differences.

### 2.5 Statistical analysis

#### 2.5.1 Quantitative data synthesis

RevMan5.3 software will be used for statistical analysis. The odds ratio (OR) and its 95% confidence interval (CI) will be used as the counting data, while the weighted mean difference (WMD) and its 95%CI will be used as the measurement data.

#### 2.5.2 Assessment of heterogeneity

The heterogeneity test will be carried out first among all studies, *I*^2^ test will be used. When *P* > 0.1 and *I*^2^ < 50%, the fixed effect model will be used; otherwise, the random effect model will be used. When the clinical heterogeneity between the 2 studies is large, only descriptive analysis will be performed.

#### 2.5.3 Publication bias

RevMan5.3 statistical software will be used to conduct qualitative analysis of publication bias in inverted funnel plots. If the funnel plot is asymmetric, there may be a publication bias in the research results.

#### 2.5.4 Subgroup analysis

If significant heterogeneity is found in our systematic review and sufficient data is available, we will conduct a subgroup analysis based on moxibustion type, moxibustion time, treatment cycle, and outcome measurement methods in the experimental and control groups.

#### 2.5.5 Sensitivity analysis

When sufficient RCTs are available, we will conduct sensitivity analysis by excluding low-quality or high-quality studies one by one according to methodological quality, sample size and missing data.

## 3 Discussion

Moxibustion is an ancient traditional Chinese medicine external therapy, which uses the warm stimulation produced by the burning of the ai material on the body surface of the relevant meridian points, so as to achieve the therapeutic effect of clinical diseases. Moxibustion therapy has the functions of warming the meridians, dispersing cold, activating blood and removing blood stasis, dredging meridians and regulating Yin and Yang.^[8,9]^ This therapy is widely used in the treatment of various systemic diseases, and its clinical efficacy has been confirmed by a large number of clinical literatures. In recent years, the clinical reports of moxibustion in the treatment of urticaria have increased year by year, but we have not seen the systematic evaluation of the effectiveness and safety of moxibustion in the treatment of urticaria. Therefore, it is necessary to systematically evaluate the treatment of urticaria by moxibustion in this study, which can provide evidence-based medicine evidence for future clinical guidance of the treatment of urticaria by moxibustion.

## Author contributions

**Data curation:** Gen Deng, Qun Wan.

**Formal analysis:** Gen Deng, Qun Wan.

**Investigation:** Qun Wan, Gen Deng.

**Methodology:** Qun Wan, Jinlong Wang.

**Project administration:** Gen Deng, Wenguo Ye.

**Software:** Qun Wan, Jinlong Wang.

**Supervision:** Wenguo Ye.

**Validation:** Wenguo Ye, Jinlong Wang.

**Visualization:** Jinlong Wang.

**Writing – original draft:** Gen Deng, Wenguo Ye.

**Writing – review & editing:** Gen Deng, Wenguo Ye.
